# Manipulation and brace fixing for the treatment of congenital clubfoot in newborns and infants

**DOI:** 10.1186/1471-2474-15-363

**Published:** 2014-10-31

**Authors:** Yuxi Su, Guoxin Nan

**Affiliations:** Ministry of Education Key Laboratory of Child Development and Disorders, Key Laboratory of Pediatrics in Chongqing, Chongqing International Science and Technology Cooperation Center for Child Development and Disorders, Department of Orthopaedics Children’s Hospital of Chongqing Medical University, Yuzhong District Zhongshan 2road 136#, Chongqing, 400014 China

**Keywords:** Clubfoot, Pirani score, Congenital, Infant, External rotation brace

## Abstract

**Background:**

As one of the most common congenital deformities in children, clubfoot has long been a challenge for orthopedic surgeons. This paper describes the experience of our team with manipulation and above-the-knee brace fixation without percutaneous Achilles tenotomy for the treatment of clubfoot in newborns and infants.

**Methods:**

In the orthopedic department of our hospital, 32 infants and newborns (56 feet) with congenital clubfoot underwent manipulation and above-the-knee brace fixation between 2008 and 2012. External rotation brace was used for 1–4 years during the night after deformity correction. Prospective follow-up for a mean duration of 29 months (range, 12–48 months) was carried out. The efficacy of the treatment was assessed by Pirani’s scoring system before and after treatment.

**Results:**

Fifty-two feet achieved a normal appearance within 3 to 6 months (average, 4.2 months) after treatment. Two patients had skin pressure sores due to improper brace care, but these healed with no scarring after timely treatment. The mean Pirani score 1 year after treatment was 0.21 ± 0.09, whereas it was 4.93 ± 1.02 before treatment (p = 0.0078). No patient required treatment with percutaneous Achilles tenotomy.

**Conclusion:**

The manipulation and brace fixation used in this study offer an effective method for correcting clubfoot deformity in newborns and infants. This treatment can be an alternative choice to percutaneous Achilles tenotomy.

**Electronic supplementary material:**

The online version of this article (doi:10.1186/1471-2474-15-363) contains supplementary material, which is available to authorized users.

## Background

**Figure 1:**
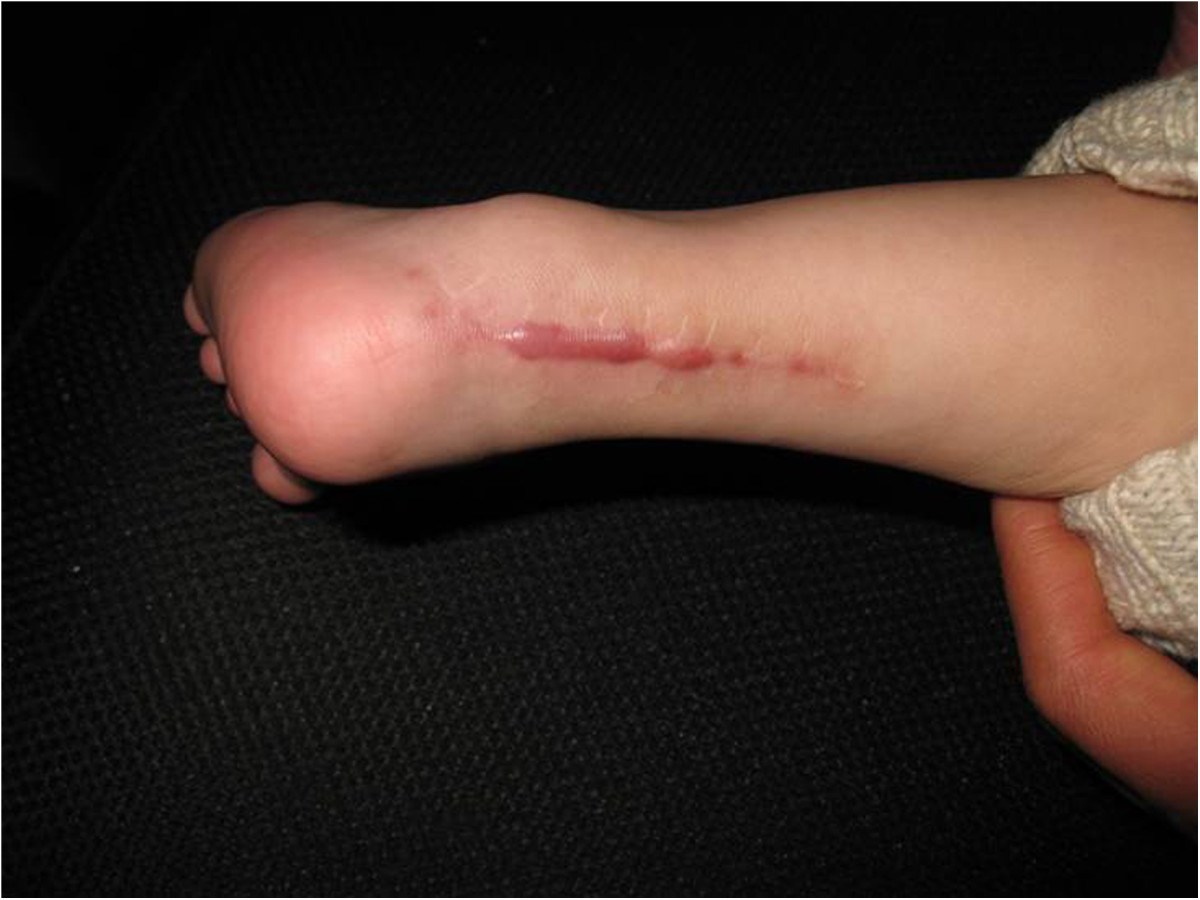
**Visible scarring can be seen 8 months after rear Achilles tendon surgery in a 1.5-year-old male patient.**

With an incidence of 1.03 per 1,000 live births, clubfoot deformity is one of the most common congenital orthopedic conditions in children [1]. The earlier treatment begins, the better the outcome will be, and many scholars have advocated initiating treatment once the diagnose is confirmed [2–4]. Treatment options include surgical and conservative treatment. Some authors report that conservative treatments other than the Ponseti method achieve lower success rates than surgery [5–7]. However, it is difficult for many parents to consent to invasive operations because of the large trauma caused by the operation, especially for patients who are not treated by the Ponseti method. Moreover, obvious scar formation can be observed on some children’s skin at the area of the rear Achilles tendon after surgery (Figure 1), and the friction in the Achilles tendon can result in pain with walking. Most surgeons agree that the initial treatment of clubfoot should be non-operative. A commonly preferred method is manipulation and application of a plaster cast at weekly intervals [8]. Ponseti developed a method that uses manipulation and casting and it does not disturb the normal physiological structure of the foot [9]. Moreover, this method is also applicable in older children. In the current study, manual correction with brace fixation was applied to treat 32 infants (a total of 56 feet) with a congenital clubfoot deformity, and its efficacy was assessed.

## Methods

### Design of the study

The complete treatment process can be divided into three phases: treatment, maintenance, and treatment. Deformity was corrected by manipulation by a therapist and parents in the first month of the treatment, and this treatment was followed by the brace fixation stage. In the maintenance phase, a brace was utilized to prevent recurrence. The maintenance phase continued for 3–4 years, and the brace was used only at night. Regular manipulation to correct the deformity was carried out by patients’ parents. Parents were informed of the treatment plan prior to the treatment to confirm that the plan could be implemented in a timely manner.

### Setting and interventions

**Figure 2:**
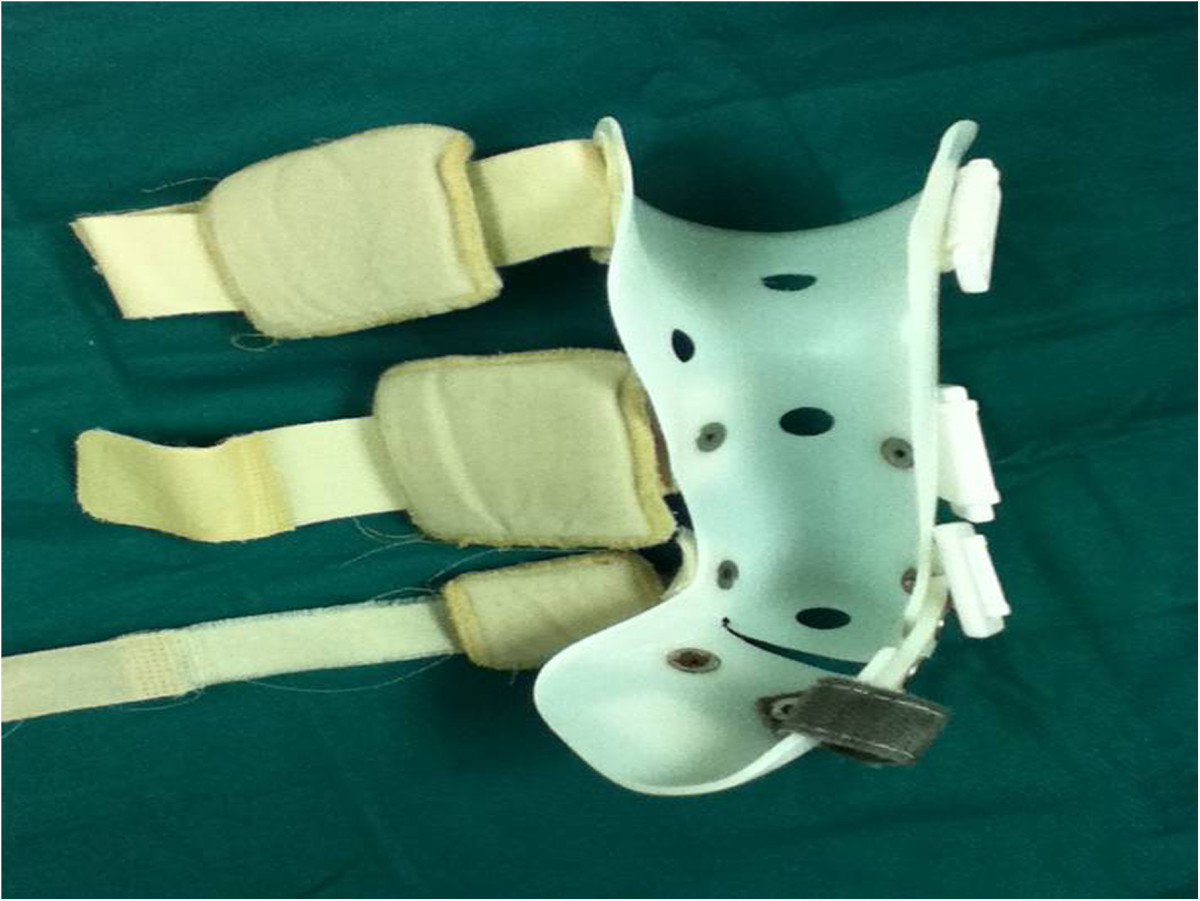
**A custom-made full-time above-the-knee brace fabricated for the left foot of a 4-month-old male patient.**

The method of manipulation was similar to the Ponseti technique. The braces were made by a technician trained by the China Disabled Persons Federation. For all patients, gypsum models were prepared first, and then braces were fabricated according to the models. The detailed technique was fully in accordance with the Ponseti method. Taking the right foot for example, the child was laid in supine position on a bed or sat on a parent’s lap. A parent held the calf, while another parent held the patient’s fore foot with their right hand, stabilizing the talus by placing the thumb of the left hand over the lateral part of the head of the talus and elevating the first ray to achieve supination of the forefoot with respect to the mid foot and hind foot. The manual correction lasted for 3–5 min and was conducted again after a short break. Manipulation continued for less than 30 min each time and was repeated 3 to 4 times each day, for a total time of at least 1.5 hours per day. One month after manipulation treatment, an above-the-knee brace was applied to patients full-time during the daytime (Figure 2). The brace was applied until the forefoot adduction and hindfoot varus deformities were corrected to a neutral or overcorrected position. This was followed by correction of the ankle equinus deformity. The skin was checked every 2 to 3 hours in the first week to prevent pressure sores. The brace was replaced once it did not match the foot size due to the rapid growth of patients. Once the deformity was corrected, the brace was used only at night time until the patients were 3–4 years old. The therapist taught the parents how to apply the manipulation technique, and the complete treatment process was described in detail in a printed manual given to the parents. All patients were scheduled to visit their doctors regularly at the outpatient clinic in order to check for residual deformities and for the assessment of brace fixation complications.

### Type of participants

This study included 32 consecutive patients with 56 clubfoot deformities who underwent manipulation and brace fixation between November 2008 and March 2012. Patients included 21 males and 11 females, and the cases included 25 right and 31 left clubfoot deformities. Deformity was bilateral in 24 patients and unilateral in 8 patients.

### Measures

All patients were assessed using Pirani’s scoring system [10] before treatment and during outpatient follow-up visits. Pirani’s scoring system has six variables (posterior crease, empty heal, equinus, reduction of the navicular bone, medial crease, and lateral curvature of the foot), and each variable is scored with 1, 0.5, or 0, with 1 indicating maximum deformity. A foot with maximum deformity has a total score of 6, whereas a normal foot has a total score of 0.

The ethics committee of the Children’s Hospital of Chongqing Medical University approved the study. The parents or guardians of the patients signed an informed consent before participation in the study and as authorization of the publication of the results and use of photographs of their children.

### Statistical analysis

The statistical analysis was performed by the first author using the SPSS 10.0 software package (SPSS, Chicago, IL, USA), and the data are given as mean ± standard deviation (SD). Inter-group data were compared with *t*-tests, and a value of p < 0.05 was considered statistically significant.

## Results

### Patient information

The average time to the initiation of treatment was 38 days after birth (0 days to 5 months). Only patients with idiopathic clubfoot deformity were included, and patients with clubfoot caused by neurological diseases, arthrogryposis multiplex congenita, and other diseases were excluded from the study. The mean initial Pirani score before treatment was 4.93 ± 1.02 (3.5–6) points (Table 1). Unfortunately, some patients were lost to follow up beyond the first year, and thus, our analysis was based on complete follow-up data for all patients for only the first year.

**Table 1:** **Pirani score and the duration of full-time brace use for patients in the two groups (g corresponds to the use of a bilateral above-the-knee supinator brace)**

Number of cases, n	Mean age pre-treatment (days)	Pirani score pre-treatment	Pirani score after 1 year	Duration of full-time brace use (months)
32 (56 feet)	38	4.93 ± 1.02	0.21 ± 0.09	4.23 + 0.89

### Treatment outcomes

**Figure 3:**
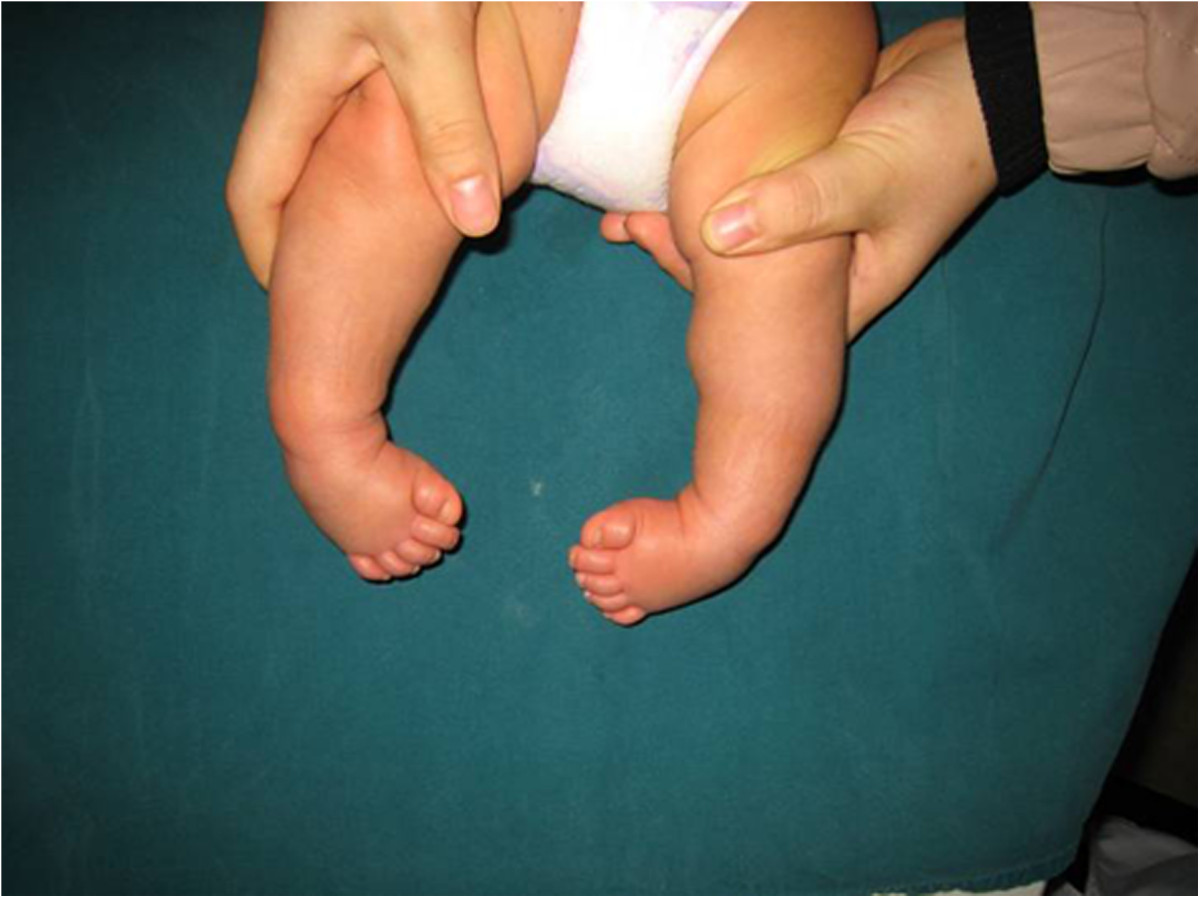
**Representative case of severe bilateral clubfoot in a 3-month-old, male patient before treatment (Pirani score = 4.6).**

**Figure 4:**
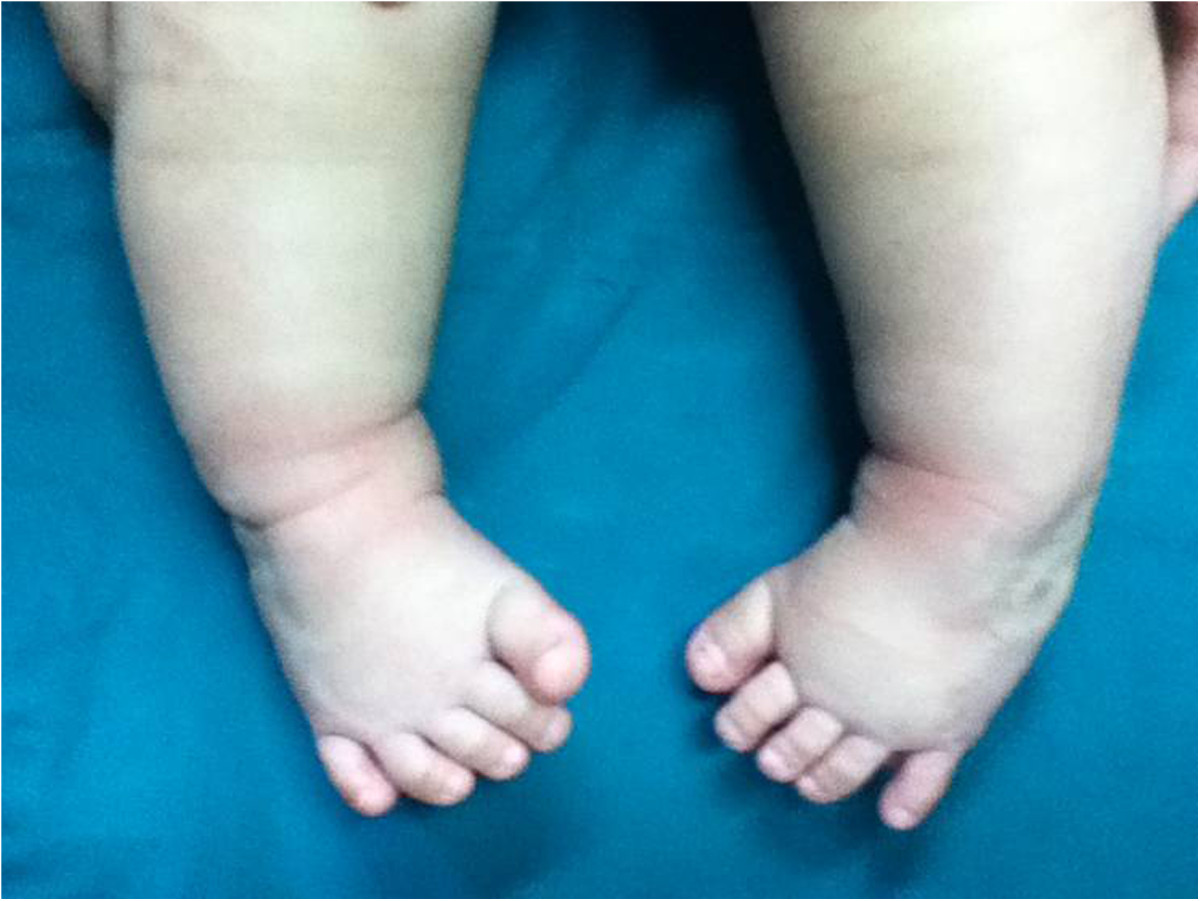
**Representative appearance after 1 month of manipulation treatment (Pirani score = 1.5).**

**Figure 5:**
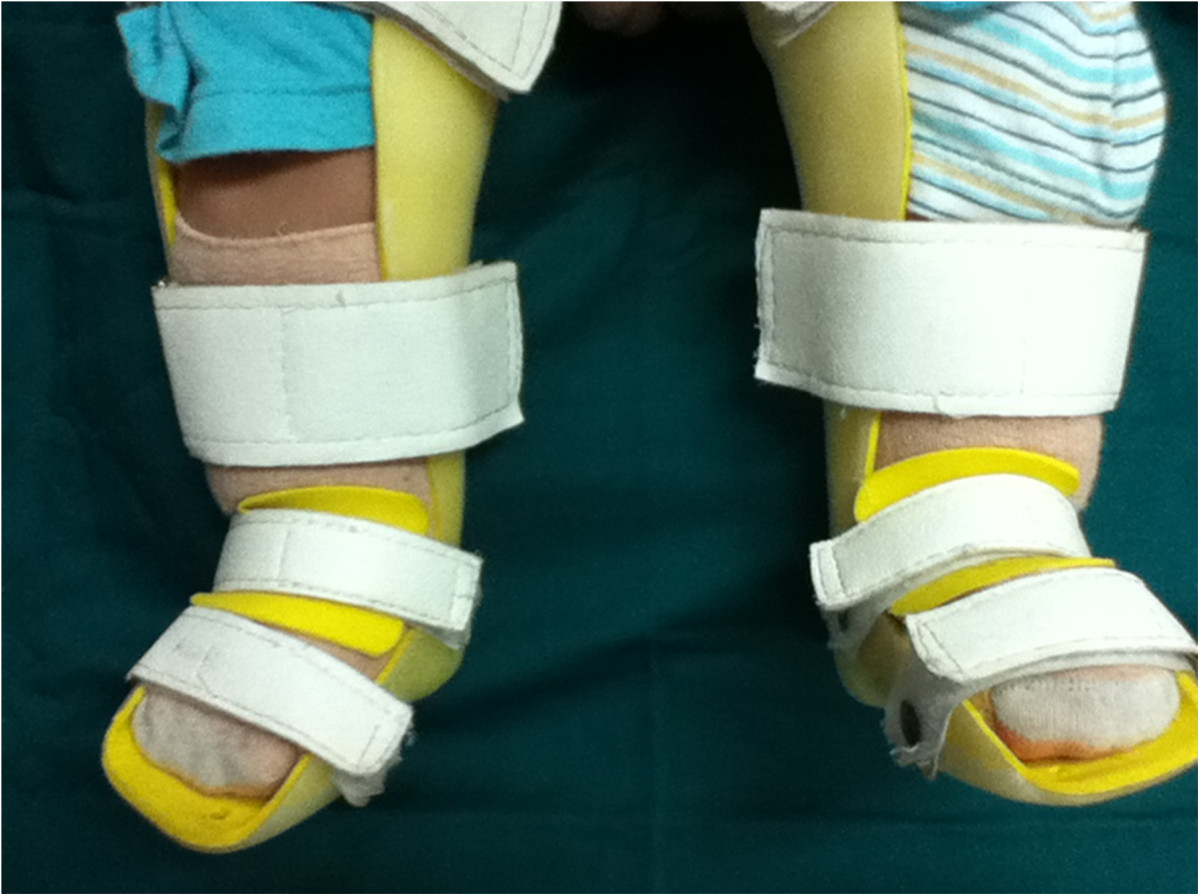
**Representative image of the final therapy outcome with correction of forefoot adduction and hindfoot varus deformities to a neutral or overcorrected position.** Nine months after treatment, the Pirani score was 0.25.

Patients were followed for 12–48 months with an average follow-up period of 29 months. The deformities were corrected to normal after treatment for 3–6 months (average, 4.23 months), and then fixation was started using a nighttime brace. Two patients had skin pressure sores due to improper brace care, but with no scarring occurred after timely treatment. Two patients with satisfactory results gave up nighttime brace fixation after being treated for 5 and 6 months. Deformity relapsed in one of these patients 3 months after stopping the treatment, as he had an Achilles tendon problem, and the other patient had no residual deformities at the time of last follow-up 1 year later. The mean Pirani score 1 year after treatment was 0.21 ± 0.09, which is significantly better than that before the treatment (p = 0.0078; Table 1). No patient required treatment with percutaneous Achilles tenotomy in this study. Photographs of representative cases are shown in Figures 3, 4, 5.

## Discussion

Although controversy remains regarding the best treatment method for clubfoot, it is widely acknowledged that early intervention is vital [2]. Generally, surgical correction of the deformity in newborns and infants is not recommended. In 1950, Ponseti developed a method that uses manipulation and casting followed by percutaneous Achilles tenotomy if equinovarus deformity persists [11–14]. Tand lead to normal appearance and function of the congenital clubfoot. The Ponseti technique does not disturb the normal physiological structure of the foot, has few long-term complications, and causes little trauma, which ensures the normal development of muscles, bones, and joints of the foot with deformity [15]. This method is also applicable in older children [16]. For evaluating the effectiveness of the treatment, we used the Pirani score for the newborns and infants rather than the Dimeglio score, which is most useful and relevant in children over 1.5 years of age [17].

The treatment method does have several disadvantages. Manual correction is not possible during the fixation period. In addition, the plaster can easily slip off of the lower limb if the physician who applies it is not experienced and appropriately trained. Most of the patients require tenotomy at a later stage. In a hot and humid climate, plaster fixation can be very uncomfortable. Moreover, bathing is not allowed during the treatment. As the infants’ skin is delicate, long-term fixation can lead to eczema and other skin diseases. Some physicians have applied a tape fixation approach to overcome such disadvantages [18]. However, tape fixation can have negative effects on skin and may even induce an allergic reaction.

The duration of follow-up for patients treated with this method has exceeded 40 years in some studies, and many patients in such studies are now living normal lives. However, the Ponseti method is associated with a relatively high rate of relapse, as was reported by the inventor himself [19, 20], and percutaneous Achilles tenotomy is needed for cases of varus deformity. In the current study, manual correction and brace fixation were applied to treat newborn and infant clubfoot patients, and satisfactory results were obtained.

Continuous force, which causes the ligament and joint capsules to relax gradually until they retain their position within the brace, is the key to manual correction. During the entire process, the patients experience almost no pain, and any lesions that develop due to treatment can be easily observed. Additionally, the braces used for fixation can be removed at any time, which makes skin care techniques much more convenient. This treatment can also be easily accepted by the patients’ parents. The principle of the treatment is that therapists and parents should strictly adhere to gradual and orderly progress of the treatment; otherwise, damage to the bones, ligaments, and joint capsules of the affected foot are likely, which will result in flat foot or rocker bottom foot deformity. The principles of this method are similar to those of the Ponseti technique. The manual correction is mainly focused on correcting the varus deformity, forefoot adduction, and hindfoot cavus first, followed by correction of the equinus. The correction degree of the brace varies in accordance with the deformity of the foot. All braces used in this study were custom-made. Considering that the patients’ limbs are too thin to fix using a brace during the first month of life, patients of this age received only massage therapy, which was followed by manipulation and above-the-knee brace fixation once the patients were old enough.

A foot abduction brace is considered mandatory to prevent relapse and is a crucial part of the Ponseti treatment [21]. The brace recommended by Ponseti is a bilateral foot abduction brace. Considering that bilateral braces restrict roll over and are uncomfortable for infants, a unilateral above-the-knee supinator brace was used in the current study to prevent relapse after the deformity was rectified. It is plausible that the brace should be used at night until pre-school. The only patient who stopped using the nighttime brace after being treated for 5 months experienced relapse 3 months later, which indicates that use of the nighttime brace is important to prevent relapse.

The key to the efficacy of this treatment method is the cooperation of the parents. Although doctors can provide individualized treatment plans for patients, parents are the executors of these plans during the treatment process. Therefore, parents should be trained to perform manipulations. The therapeutic schedule should be carefully designed to establish confidence in parents to cooperate with the treatment. Parents must be informed to watch children closely and to remove the brace to check for possible compression injury on the skin during the initial period of brace fixation. In the current study, two patients experienced skin pressure sores in the treatment group but no scarring occurred after timely treatment.

Treatment by manipulation of the brace fixation is safe, comfortable, and conducive to observation and skin care. However, the present study did not include conservative treatment for older children, for which further research is still needed. Some patients may still require tenotomy at a longer follow-up time [22]. We believe that the deformity can be improved with this treatment as long as the feet have begun bearing weight, and even if the result is not satisfactory, the treatment may create better conditions for further surgical treatment.

## Conclusion

The results of this study indicate that manipulation and brace fixing can be an effective treatment for congenital clubfoot deformity in newborns and infants. However, a longer follow-up period may be needed to evaluate the long-term efficacy of this method.

## Authors’ original submitted files for images

Below are the links to the authors’ original submitted files for images. Authors’ original file for figure 1 Authors’ original file for figure 2 Authors’ original file for figure 3 Authors’ original file for figure 4 Authors’ original file for figure 5
