# Supplementary figures and images for: Manipulation and brace fixing for the treatment of congenital clubfoot in newborns and infants
Source: BMC Musculoskelet Disord. 2014 Oct 31;15:363. doi: 10.1186/1471-2474-15-363 (PMC4232732; doi:10.1186/1471-2474-15-363)

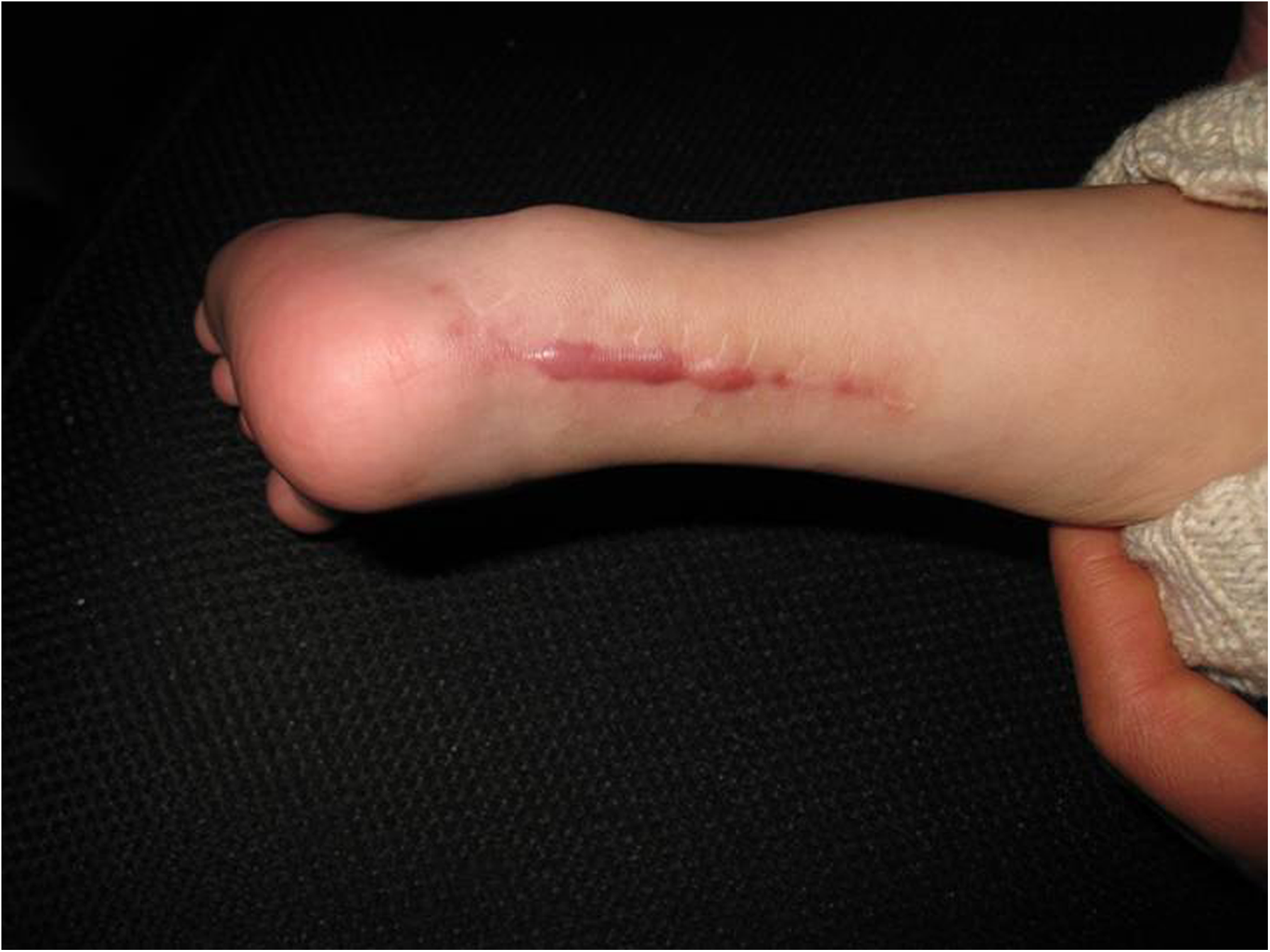

Supplement: Supplementary file 1 — Authors’ original file for figure 1 [file 12891_2014_2304_MOESM1_ESM.tif]

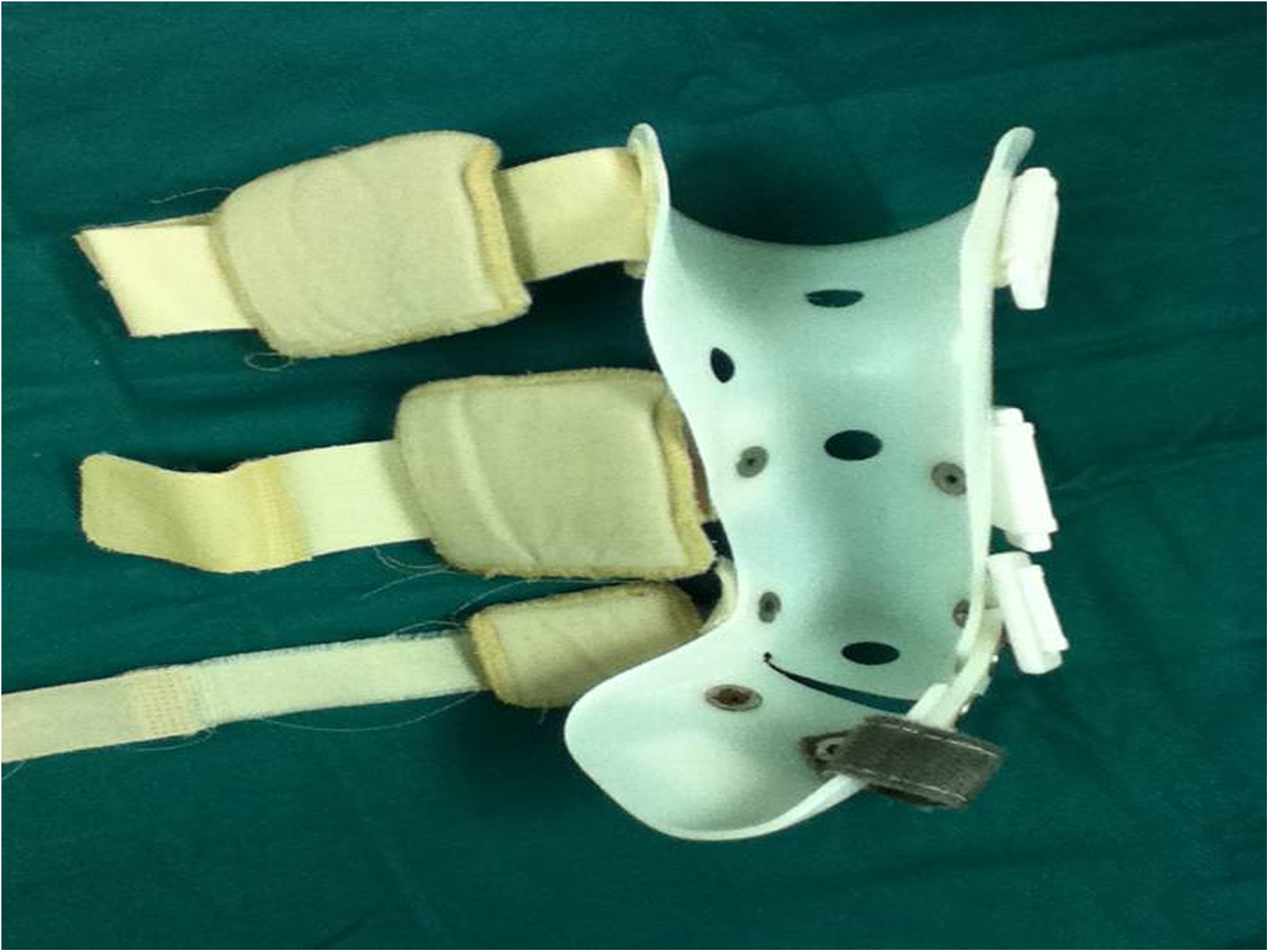

Supplement: Supplementary file 2 — Authors’ original file for figure 2 [file 12891_2014_2304_MOESM2_ESM.tif]

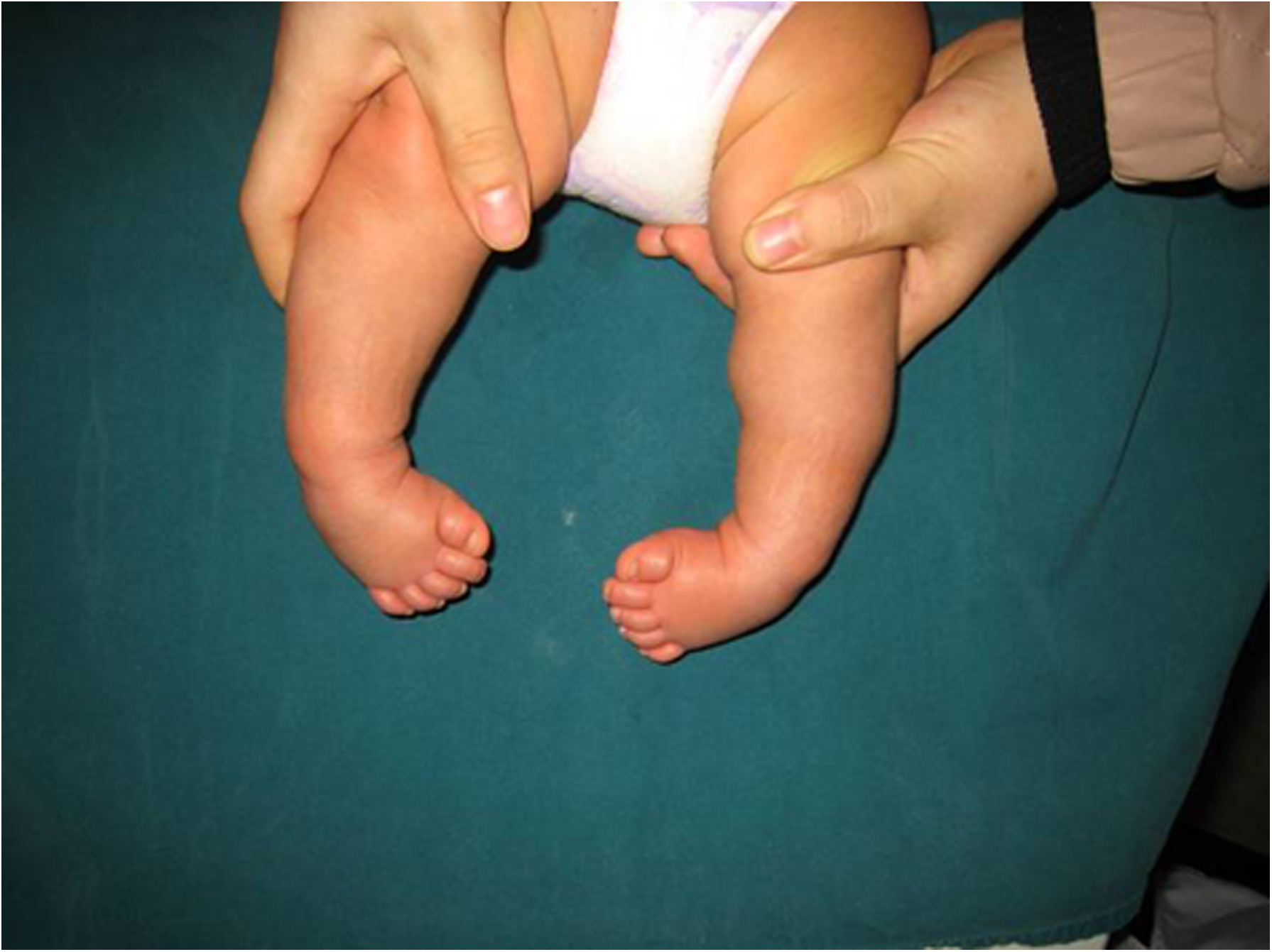

Supplement: Supplementary file 3 — Authors’ original file for figure 3 [file 12891_2014_2304_MOESM3_ESM.tif]

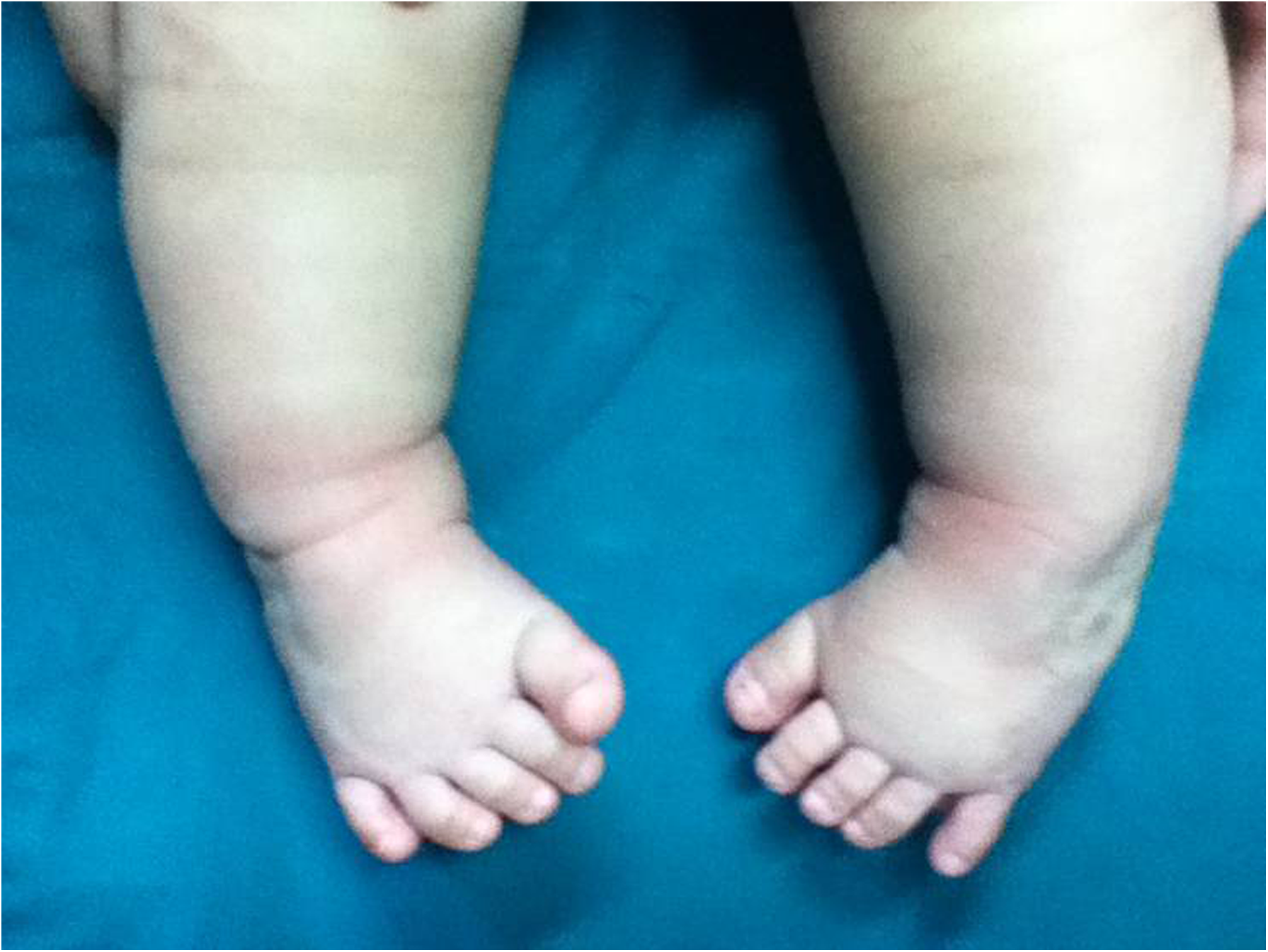

Supplement: Supplementary file 4 — Authors’ original file for figure 4 [file 12891_2014_2304_MOESM4_ESM.tif]

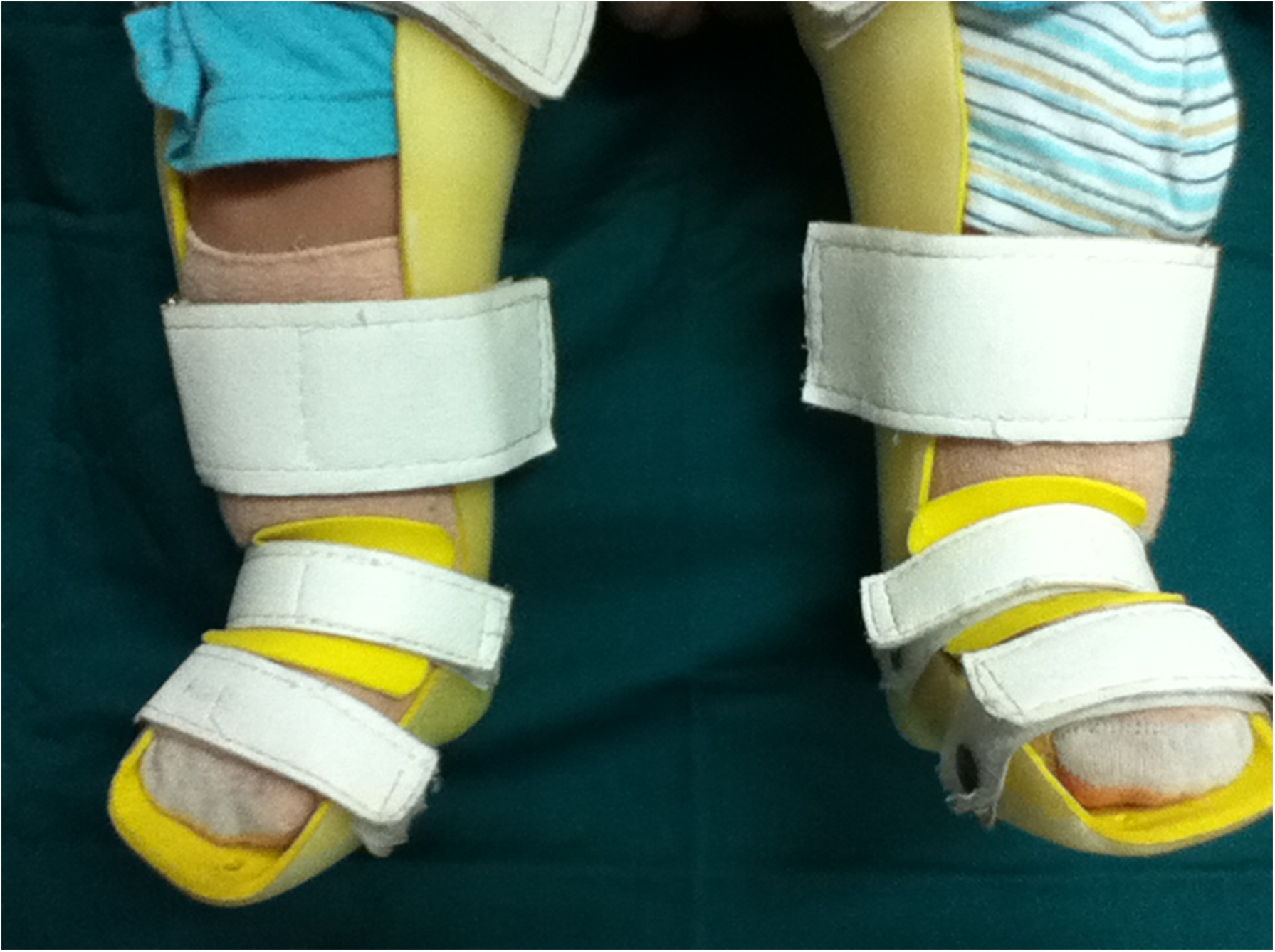

Supplement: Supplementary file 5 — Authors’ original file for figure 5 [file 12891_2014_2304_MOESM5_ESM.tif]
